# Split Cyclin-Dependent Kinase 4/6–Retinoblastoma 1 Axis in Pancreatic Cancer

**DOI:** 10.3389/fcell.2020.602352

**Published:** 2020-11-12

**Authors:** Xing Huang, Gang Zhang

**Affiliations:** Zhejiang Provincial Key Laboratory of Pancreatic Disease, School of Medicine, Zhejiang University, Hangzhou, China

**Keywords:** CDK4/6, combination therapy, pancreatic cancer, precise treatment, prognosis, Rb1

## Abstract

Drugs targeting the cyclin-dependent kinase 4/6 (CDK4/6)–retinoblastoma 1 (RB1) axis have shown efficacy against multiple solid cancers, but their therapeutic potential in pancreatic cancer remains poorly defined. A recent report proposed that a “tailored” combination of first-line and second-line CDK4-targeting drugs would hold promise for pancreatic cancer treatment. Indeed, this therapeutic strategy exhibited significantly suppressive effects on pancreatic cancer patient-derived cell lines and tumor tissue *in vitro*. However, the study neglected immune involvement and the influence of CDK6 and RB1 in CDK4 inhibition-based treatment. Herein, we reveal multiple new facets of the CDK4/6–RB1 axis in pancreatic cancer, highlighting the complexity of this signaling axis for future prognostic and therapeutic targeting.

## Introduction

Pancreatic ductal adenocarcinoma (PDA) is the most prevalent pathohistological type of pancreatic cancer and is characterized by high lethality rates (Kleeff et al., 2016). Mutations in multiple tumor suppressor genes contribute to the tumorigenesis and progression of PDA, including but not limited to *CDKN2A* (Collisson et al., 2019). As a Polycomb repressed cell cycle checkpoint gene, *CDKN2A* encodes P16^INK4A^ that binds to cyclin D-cyclin-dependent kinase 4/6 (CDK4/6) to prevent retinoblastoma 1 (RB1) phosphorylation (Rubin, 2013). In its hypo-phosphorylated state, RB1 inhibits cell cycle progression through binding and inhibiting the E2F family of transcription factors (van den Heuvel and Dyson, 2008). Mutations in *CDKN2A* result in sustained RB1 phosphorylation, dysregulated cell cycle progression, and tumor cell proliferation. Recently, Chou et al. (2018) reported a “tailored” combination of first-line and second-line CDK4-targeting drugs for PDA therapy, guided by the predictive marker RB1. Although this strategy showed translational potential, genomic analyses suggest that the conventional CDK4/6–RB1 signaling pathway is divergent in pancreatic cancer, questioning the efficacy of this therapy.

## Methods

Data on the prognostic potential of CDK4/6 and RB1 in pancreatic cancer were collected from The Cancer Genome Atlas (TCGA)^1^ database. The Tumor and Immune System Interaction Database^2^ was used to examine the correlation between the abundance of immunomodulators and the expression of investigative genes. Gene Expression Profiling Interactive Analysis 2^3^ was used to calculate the prognostic index, including gene expression level and its correlation to patient survival. cBioPortal for Cancer Genomics^4^ was used to visualize and compare gene alterations. A one-way ANOVA was used to analyze the differential expression of CDK4/6 and RB1 and the abundance of immunomodulators. Genes with |log2FC| values > 1 and *q* values < 0.01 were considered statistically significant. Overall survival (OS) analyses of CDK4/6 and RB1 were assessed using the Kaplan–Meier method, with a 50% (median) cutoff for both low and high expression groups. The log-rank test (the Mantel–Cox test) was used for hypothesis testing, and the Cox proportional hazard regression model was used to calculate the hazard ratio. *P*-values ≤ 0.05 were used as a threshold when ranking the data. Spearman correlation analysis was used to analyze the correlation between the pair-wise gene expression of CDK4 and a range of immunomodulators. *P*-values < 0.01 were considered as significant. CDK4-related immunomodulators were collected, according to Charoentong et al.

## Results

### Cyclin-Dependent Kinase 4 Correlates With Immune Regulation in Pancreatic Cancer

**FIGURE 1:**
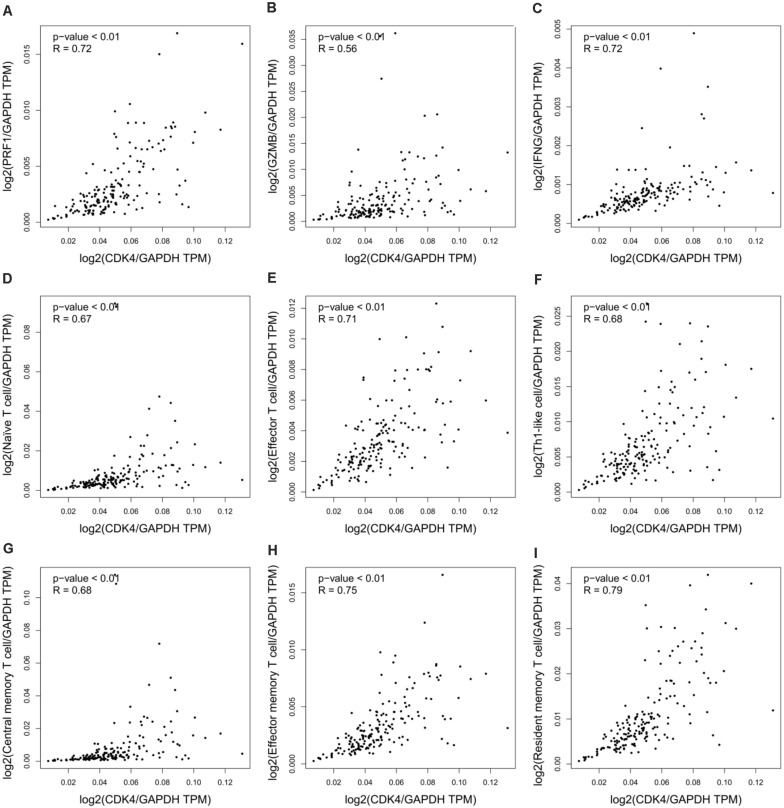
CDK4 correlates with immune regulation in pancreatic cancer. **(A–C)** Correlation analysis of CDK4 and the immune effector molecules PRF1 **(A)**, GZMB **(B)**, and IFNG **(C)** in PAAD. **(D–I)** Correlation analysis of CDK4 and immune cell signatures in PAAD, including naïve T cell signatures **(D)**, effector T cell signatures **(E)**, Th1-like cell signatures **(F)**, central memory T cell signatures **(G)**, effector memory T cell signatures **(H)**, and resident memory T cell signatures **(I)**. *P*-values ≤ 0.05 were considered statistically significant.

Despite the importance of immune activation for cancer therapy (Sanmamed and Chen, 2018), experimental models for PDA therapeutics often fail to recapitulate host defense systems. Chou et al. (2018) used patient-derived tumor xenografts, which must be implanted into immunodeficient mice, to analyze the therapeutic efficacy of CDK4 inhibition on pancreatic cancer. This inevitably bypasses any immune involvement and fails to recapitulate the tumor immune microenvironment. Moreover, previous studies have validated the role of CDK4 in the regulation of cancer immunity and immunotherapy. Goel et al. (2017) reported that CDK4/6 inhibition triggers anticancer immune responses in breast cancer, and Zhang et al. (2018) showed that cyclin D-CDK4 kinase destabilizes programmed death-ligand 1 via the Cul3^SPOP^ complex to control cancer immunosurveillance in melanoma cancer. To determine the immunologic relevance of CDK4 in pancreatic cancer, we integrated all relevant datasets in the TCGA database for bioinformatics analyses. In pancreatic adenocarcinoma (PAAD), the results showed a positive correlation between CDK4 and the immunomodulatory system, including the immune effector molecules PRF1, GZMB, and IFNG (Figures 1A–C), as well as immune cells (naïve T cells, effector T cells, Th1-like cells, central memory T cells, effector memory T cells, and resident memory T cells) (Figures 1D–I). These data suggest that the outcomes of CDK4-based PDA therapy in immunocompromised models have limited significance to the clinical treatment of PDA.

### Opposite Prognoses of Cyclin-Dependent Kinase 4 and 6 in Pancreatic Cancer

**FIGURE 2:**
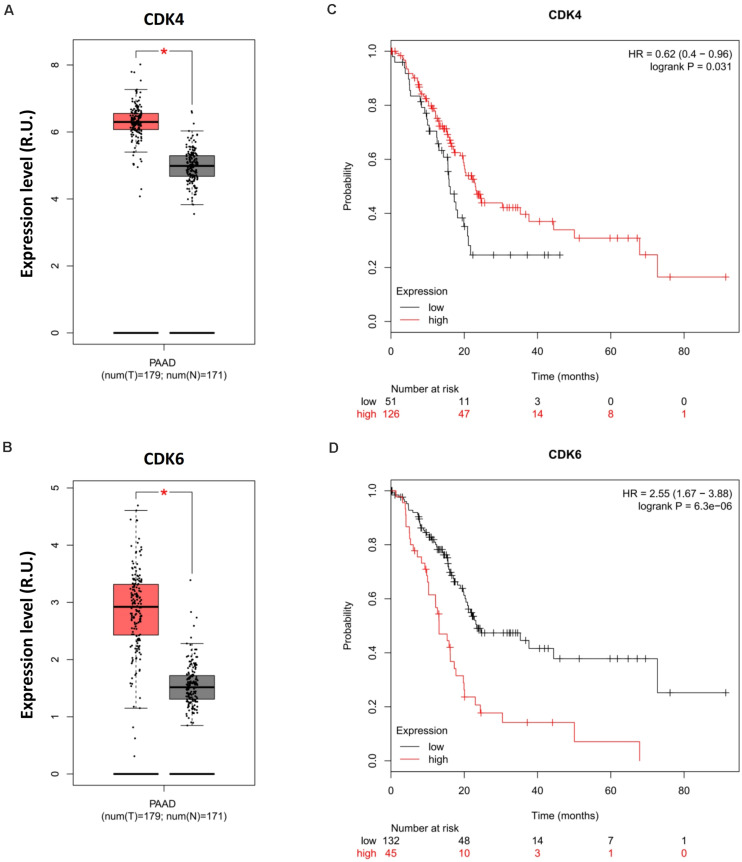
Opposite prognoses of CDK4 and CDK6 in pancreatic cancer. Differential expression analysis of **(A)** CDK4 and **(B)** CDK6 in PAAD. OS analysis of **(C)** CDK4 and **(D)** CDK4 in PDA. *P*-values ≤ 0.05 were considered statistically significant. ^∗^*p*≤0.01.

Chou et al. (2018) recently highlighted the potential of CDK4 inhibitors as a targeted therapy for pancreatic cancer. However, Palbociclib (PD-0332991, Pfizer), the major CDK4-targeted drug used in the study, is a small molecule inhibitor designed to block the activity of both CDK4 and CDK6 to promote cytostasis (O’Leary et al., 2016). The concomitant effects of this therapy on CDK6 in more relevant physiological models of pancreatic cancer can, therefore, not be discounted. In light of this, we analyzed relevant genomic data and observed the opposite effects of CDK4 and CDK6 on pancreatic cancer prognosis. The expression levels of CDK4 and CDK6 were highly upregulated in PAAD compared with normal tissue (Figures 2A,B); however, CDK4 promoted rather than suppressed the OS of PDA patients (Figure 2C), whereas high CDK6 expression was associated with poor survival (Figure 2D). This phenotype implicates CDK6 as opposed to CDK4 for the poor prognosis of PDA in the clinic and questions the therapeutic benefits of Palbociclib in PDA therapy due to its synergistic effects on CDK4 and CDK6. In light of these data, patient-derived primary cell lines that genetically lack CDK4 and CDK6 should be generated to investigate their significance during Palbociclib treatment and exclude off-target effects. Of note, the combination of palbociclib and gemcitabine was investigated in the study as mentioned earlier, but the therapeutic efficacy of gemcitabine has been reported to be influenced by tumor immune microenvironment during pancreatic cancer therapy (Halbrook et al., 2019), which further highlights the importance of appropriate models in evaluating the CDK4/6-targeted combinational strategy.

### Retinoblastoma 1 Is Coupled to Cyclin-Dependent Kinase 4 and Poor Prognosis in Pancreatic Cancer

**FIGURE 3:**
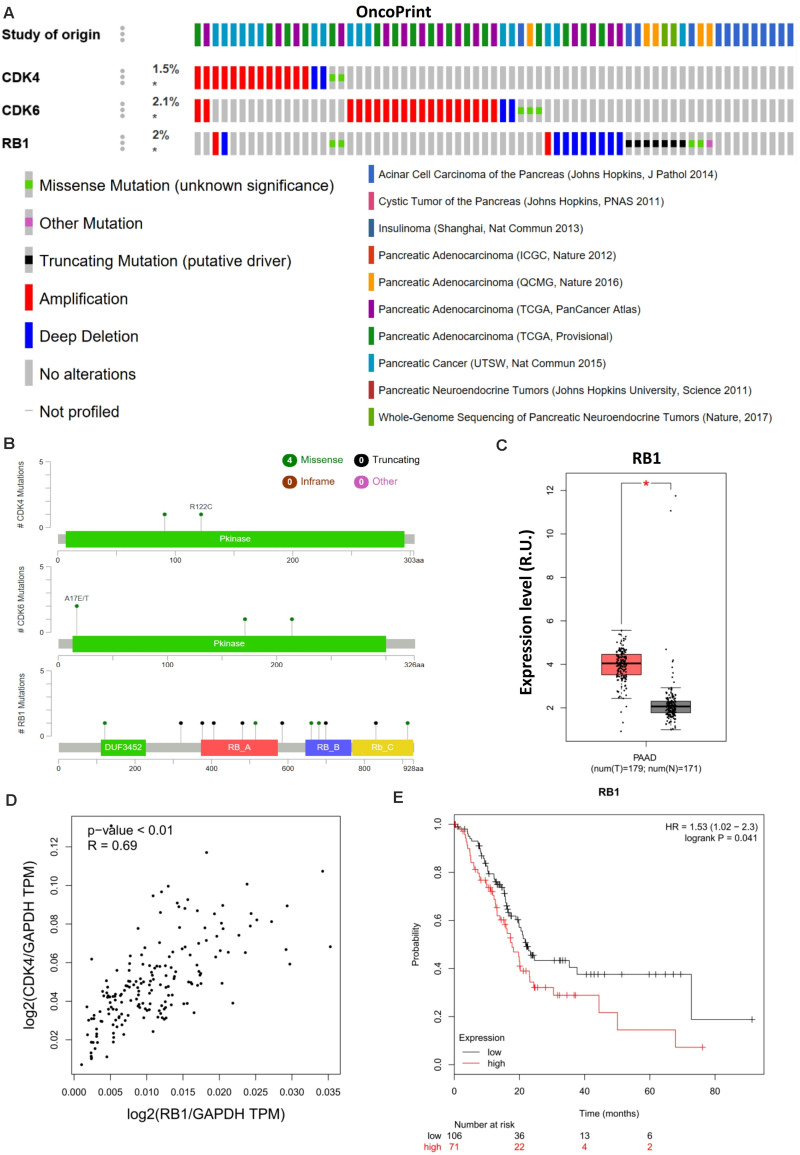
RB1 couples with CDK4 and is related to poor prognosis in pancreatic cancer. **(A)** Landscape of the CDK4/6–RB1 axis in pancreatic cancer. Compact visualization of cases with different genetic alterations, including fusions, amplification, deletions, truncating mutations, and missense mutations. **(B)** Detailed mutational analysis of CDK4, CDK6, and RB1 in pancreatic cancer. **(C)** Differential expression analysis of RB1 in PAAD. **(D)** Correlation analysis of RB1 and CDK4 in PAAD. **(E)** OS analysis of RB1 in PAAD. *P*-values ≤ 0.05 were considered statistically significant. ^∗^*p*≤0.01.

Cyclin-dependent kinase 4/6 controls the downstream transcriptional signals that regulate cancer cell proliferation through the phosphorylation of their canonical substrate RB1. CDK4/6–phosphorylated RB1 has been shown to promote anticancer immunity by inhibiting nuclear factor-κB activation and programmed death-ligand 1 expression (Jin et al., 2019), further indicating the immunological relevance of palbociclib during cancer treatment. Moreover, aberrant CDK4/6-RB1 signaling has been reported in a range of cancers. Such alterations regulate immune evasion and immunotherapy (Hutcheson et al., 2015). Genomic analyses indicated that a similar phenotype exists in pancreatic cancer in which CDK4 regulates RB1 activation (Figures 3A,B). However, Chou et al. (2018) used only 19 whole-genome-sequenced pancreatic cancer patient-derived primary cell lines to exclude the potential impact of RB1 mutations *in vivo*. Although RB1 is highly expressed (Figure 3C) and positively related to CDK4 (Figure 3D) in PAAD, higher expression of RB1 was related to poor prognosis (Figure 3E), contradictory to the previous conclusions. The differential outcomes between genomic analysis and immunohistochemistry staining were caused by the failure to account for immune function and/or alterations in RB1. Hence, the phosphorylation of RB1 as a biomarker when predicting the therapeutic sensitivity of CDK4/6 inhibition requires further clinical and genetic analysis.

## Conclusion

In summary, we highlight the importance of CDK4 for immune regulation. We further show that elevated CDK4 expression is a favorable prognostic index for cancer patients instead of high CDK6 expression that is close to poor prognosis. RB1 was positively related to CDK4 but linked to a poorer prognosis. These data suggest that the direct anti-tumor effects of CDK4/6 inhibition in xenografts fail to reflect their efficacy in pancreatic cancer patients. The utility of targeting CDK4/6 as a strategy for targeted-immunotherapy in pancreatic cancer now warrants further exploration.

## Data Availability Statement

The original contributions presented in the study are included in the article/supplementary material, further inquiries can be directed to the corresponding author.

## Author Contributions

XH conceived this manuscript, conducted the analyses, and interpreted the data. XH and GZ drafted, discussed, and revised the manuscript. Both authors read and approved the final version.

## Conflict of Interest

The authors declare that the research was conducted in the absence of any commercial or financial relationships that could be construed as a potential conflict of interest.

## Footnotes

1 http://cancergenome.nih.gov

2 http://cis.hku.hk/TISIDB

3 http://gepia2.cancer-pku.cn

4 http://www.cbioportal.org

## Abbreviations

PAAD: pancreatic adenocarcinoma
PDA: pancreatic ductal adenocarcinoma
RB1: retinoblastoma 1
TCGA: The Cancer Genome Atlas.
